# Red Blood Cell Distribution Width as a Predictor of 28‐Day Mortality in Critically Ill Patients With Alcohol Use Disorder

**DOI:** 10.1111/acer.14483

**Published:** 2020-11-16

**Authors:** Lin Liao, Liao Pinhu

**Affiliations:** ^1^ From the School of Medicine Guangxi University Nanning China

**Keywords:** Alcohol Use Disorder, Critically Ill Patients, Red Blood Cell Distribution Width, 28‐Day Mortality

## Abstract

**Background:**

Patients with alcohol use disorder (AUD) are common attendees of the intensive care unit (ICU). Early assessment of the prognosis for critically ill patients with AUD is conducive for formulating comprehensive treatment measures and improving survival rates. The purpose of this study was to explore the predictive value of red blood cell distribution width (RDW) for 28‐day mortality in critically ill patients with AUD.

**Methods:**

2,884 patients with AUD were recruited retrospectively. Data from the MIMIC‐III database were collected and analyzed. A receiver operating characteristic (ROC) curve was used to determine the optimal cutoff value of RDW. The Kaplan–Meier method and Cox regression models were used to evaluate prognostic factors.

**Results:**

Of the 2,884 patients, there were 344 nonsurvivors (11.9%). The nonsurvivors had a higher RDW than the survivors (*p* < 0.001). According to ROC curve analysis, the area under the curve predicted by RDW for 28‐day mortality was 0.728 (95% CI, 0.700 to 0.755) and the optimal cutoff value was 15.45% (sensitivity: 67.2%; specificity: 67.3%). Length of stay in ICU, length of stay in hospital, in‐hospital mortality, and 28‐day mortality in patients with an RDW > 15.45% were significantly higher than in those with an RDW ≤ 15.45% (*p* < 0.001). Cox regression analysis showed that an RDW > 15.45% was an independent prognostic indicator for 28‐day mortality in critically ill patients with AUD (HR = 1.964, 95% CI: 1.429 to 2.698).

**Conclusions:**

High RDW was associated with increased short‐term mortality risks in critically ill patients with AUD.

Alcohol is the most common and abused addictive substance in the general population. Harmful use of alcohol not only damages the body, but also causes mental disorders and social harm (Walls et al., 2020). It is estimated that 28 million deaths worldwide were attributed to alcohol consumption in 2016 (Collaborators GBDA, 2018). In addition, as the level of global development increases, the number of people affected by alcohol use has also been shown to rise (Jernigan and Babor, 2015). The diagnoses of alcohol abuse and alcohol dependence were merged in the *Diagnostic and Statistical Manual of Mental Disorders*, Fifth Edition (DSM‐5), and are now collectively referred to as alcohol use disorder (AUD; Francesmonneris et al., 2013). AUD is common in critically ill patients, with 16 to 31% of patients admitted to the intensive care unit (ICU) being diagnosed with having AUD (Dixit et al., 2016).

When compared with patients who do not drink, those with AUD have a higher risk of serious illness, a greater risk of comorbidities, and a worse prognosis (Mehta, 2016). Therefore, it is extremely important to evaluate the disease severity and prognosis of critically ill patients with AUD at an early stage and take timely intervention measures. Red blood cell distribution width (RDW) is a parameter which reflects the heterogeneity of the volume of red blood cells and is often used in the differential diagnosis of anemia (Salvagno et al., 2015). In recent years, studies have found that high RDW is closely related to the prognosis of many severe diseases including sepsis, severe pancreatitis, acute kidney injury, and ARDS (Jia et al., 2020; Jo et al., 2013; Wang et al., 2019; Zhang et al., 2019). However, there are no reports examining the relationship between RDW and prognosis in critically ill patients with AUD. The purpose of this study was to evaluate the predictive value of RDW in the short‐term prognosis of critically ill patients with AUD.

## Materials and Methods

### Data Source

The data analyzed in this study were obtained from the Medical Information Mart for Intensive Care III (MIMIC‐III) database (version 1.4). MIMIC‐III is a public database jointly developed by the Laboratory for Computer Physiology at Massachusetts Institute of Technology (MIT), Beth Israel Deaconess Medical Center, and Philips Healthcare. The database has records regarding the demographics, vital signs, and survival data of more than 40,000 patients that have been in the ICU of Beth Israel Deaconess Medical Center. After removing patient identification information, data are freely accessible to all researchers and available from the PhysioNet website (http://www.physionet.org; Johnson et al., 2016). The use of the database in this study was approved by the Institutional Review Boards of Beth Israel Deaconess Medical Center and MIT (certification number 30165505), and an exemption for informed consent was obtained.

### Case Inclusion Criteria

ICD‐9 codes (291.X, 291.XX, 303.XX, 303.XX, 305.XX, 535.3X, 357.5, 425.5, and 571.0 to 571.3 where X stands for wildcards) were used to identify AUD patients. Exclusion criteria were as follows: younger than 18 years of age, not the first time entering the ICU, stayed in the ICU for less than 24 hours, and where RDW results were missing or cases where more than 5% personal data were missing.

### Data Extraction

Demographic characteristics, comorbidities, as well as laboratory parameters, systolic blood pressure, respiratory rate, heart rate, sequential organ failure assessment (SOFA) scores, and simplified acute physiology score II (SAPS II) scores taken within the first 24 hours after entering the ICU were collected. Extracted comorbidities included congestive heart failure, hypertension, liver disease, electrolyte disorders, coagulopathy, anemia, and chronic pulmonary disease. Laboratory parameters included white blood cell (WBC) and platelet counts, mean corpuscular volume (MCV) and RDW, hemoglobin and glucose levels, and the activities of alanine aminotransferase and aspartate aminotransferase (AST). Model for end‐stage liver disease (MELD) was calculated as follows: MELD = 0.957 × ln(creatinine mg/dl) + 0.378 × ln(bilirubin mg/dl) + 1.120 × ln(INR) + 0.643 (Kamath et al., 2001). The study endpoint was defined as the 28‐day all‐cause mortality from the date of admission to the ICU. The means or medians were used for missing data measurements.

### Statistical Analysis

Continuous variables were reported as median (interquartile range [IQR]) and compared using Mann–Whitney test. Categorical variables were reported as numbers and percentages and compared using chi‐square test. A receiver operating characteristic (ROC) curve was drawn to evaluate the predictive ability of RDW for 28‐day mortality and to determine the optimal cutoff value. The patients were divided into a high RDW group and a low RDW group, according to the optimal cutoff value of RDW. The Kaplan–Meier method was used for survival analysis, and the log‐rank test was used to compare survival rates between groups. Univariate and multivariate Cox regression models were used to assess risk factors associated with 28‐day mortality. *p* < 0.05 was considered statistically significant. All statistical analysis was performed using SPSS 22.0 software, and the graphs were drawn using GraphPad Prism 6.0 software.

## Results

### Patient Characteristics

A total of 2,884 AUD patients were studied, including 683 (23.7%) females and 2,201 (76.3%) males. The median age was 52.6 years (IQR: 43.6 to 62.2 years), and the majority of patients were Caucasian (71.1%). The total number of deaths was 344 (11.9%). The most common comorbidities were hypertension (39.3%), electrolyte disorders (38.1%), and liver disease (33.6%). The nonsurvivors had a higher RDW than the survivors (*p* < 0.001). Table 1 summarizes the baseline characteristics of all the study patients.

**Table 1:** Baseline Characteristics of Critically Ill Patients With AUD

	All (*n* = 2,884)	Survivors (*n* = 2,540)	Nonsurvivors (*n* = 344)	*p* value
Age, year	52.6 (43.6 to 62.2)	52.0 (42.8 to 61.4)	57.6 (49.8 to 67.5)	<0.001
Gender, *n* (%)				0.455
Female	683 (23.7%)	596 (23.5%)	87 (25.3%)	
Male	2,201 (76.3%)	1,944 (76.5%)	257 (74.7%)	
Ethnicity, *n* (%)				0.008
White	2,050 (71.1%)	1,814 (71.4%)	236 (68.6%)	
Black	216 (7.5%)	200 (7.9%)	16 (4.7%)	
Others	618 (21.4%)	526 (20.7%)	92 (26.8%)	
SBP, mm Hg	120.4 (108.8 to 133.4)	121.3 (110 to 133.9)	111.3 (100.3 to 126.9)	<0.001
Heart rate, beats/minute	88.7 (78.4 to 99.7)	88.6 (78.2 to 99.3)	90.3 (79.1 to 102.9)	0.026
Respiratory rate, beats/minute	18.5 (16.3 to 21.2)	18.3 (16.3 to 21)	19.9 (17.1 to 23.3)	<0.001
Scoring systems				
SOFA	4.0 (2.0 to 7.0)	4.0 (2.0 to 6.0)	9.0 (5.3 to 12.0)	<0.001
SAPS II	29.0 (22.0 to 40.0)	28.0 (20.0 to 37.0)	46 (36.3 to 57.0)	<0.001
MELD	11.0 (8.0 to 20.0)	11.0 (8.0 to 20.0)	10.0 (7.0 to 20.0)	0.339
Comorbidities, *n* (%)				
Congestive heart failure	398 (13.8%)	337 (13.3%)	61 (17.7%)	0.024
Hypertension	1,133 (39.3%)	1,003 (39.5%)	130 (37.8%)	0.545
Liver disease	970 (33.6%)	752 (29.6%)	218 (63.4%)	<0.001
Electrolyte disorders	1,099 (38.1%)	896 (35.3%)	203 (59.0%)	<0.001
Coagulopathy	626 (21.7%)	487 (19.2%)	139 (40.4%)	<0.001
Anemia	2,296(79.6%)	1,998 (78.7%)	298 (86.6%)	<0.001
Chronic pulmonary disease	450 (15.6%)	389 (15.3%)	61 (17.7%)	0.246
Laboratory parameters				
WBC, 10^9^/l	11.4 (8.0 to 16.2)	11.2 (7.9 to 15.9)	13.8 (9.4 to 19.7)	<0.001
Hemoglobin, g/dl	10.7 (9.0 to 12.4)	10.8 (9.1 to 12.5)	9.8 (8.5 to 11.4)	<0.001
MCV, fl	93.0 (89.0 to 99.0)	93.0 (89.0 to 98.0)	98.0 (92.0 to 104.0)	<0.001
RDW, %	14.6 (13.5 to 16.5)	14.4 (13.4 to 16.1)	16.7 (14.8 to 18.7)	<0.001
Platelet, 10^9^/l	157.0 (98.0 to 223.0)	162.0 (103.0 to 226.0)	107.0 (61.3 to 194.5)	<0.001
ALT, IU/l	68.0 (27.0 to 200.6)	70.0 (27.0 to 200.6)	56.0 (28.3 to 200.6)	0.211
AST, IU/l	123.0 (47.0 to 331.3)	119.0 (45.0 to 331.3)	134.5 (62.0 to 331.3)	0.204
Glucose, mg/dl	141.0 (116.0 to 182.8)	140.0 (115.0 to 180.8)	148.0 (120.0 to 199.5)	0.006
ICU LOS, day	2.8 (1.7 to 6.0)	2.7 (1.7 to 5.6)	4.2 (2.2 to 8.5)	<0.001
Hospital LOS, day	8.2 (4.4 to 15.5)	8.2 (4.5 to 16)	8.1 (4.0 to 13.3)	0.004

Continuous covariates are reported as median (interquartile range) and categorical variables as *n* (%).

ALT, alanine aminotransferase; AST, aspartate aminotransferase; AUD, alcohol use disorder; ICU, intensive care unit; LOS, length of stay; MCV, mean corpuscular volume; MELD, Model for End to stage Liver Disease; RDW, red cell distribution width; SAPS II, Simplified Acute Physiology Score II;SBP, systolic blood pressure; SOFA, Sequential Organ Failure Assessment; WBC, white blood cell.

### The Value of 28‐Day Mortality Predicted by RDW in AUD Patients

**Fig. 1:**
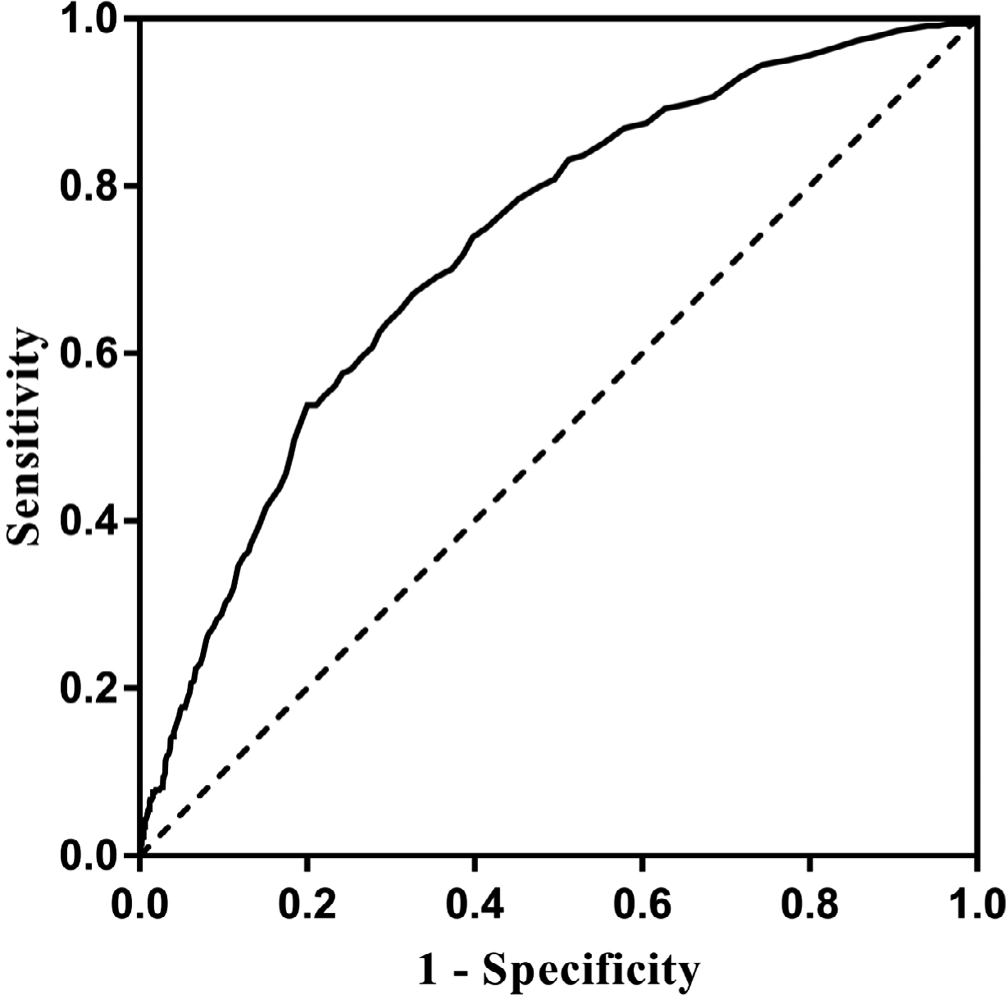
The role of the RDW ROC curve in determining 28‐day mortality.

**Fig. 2:**
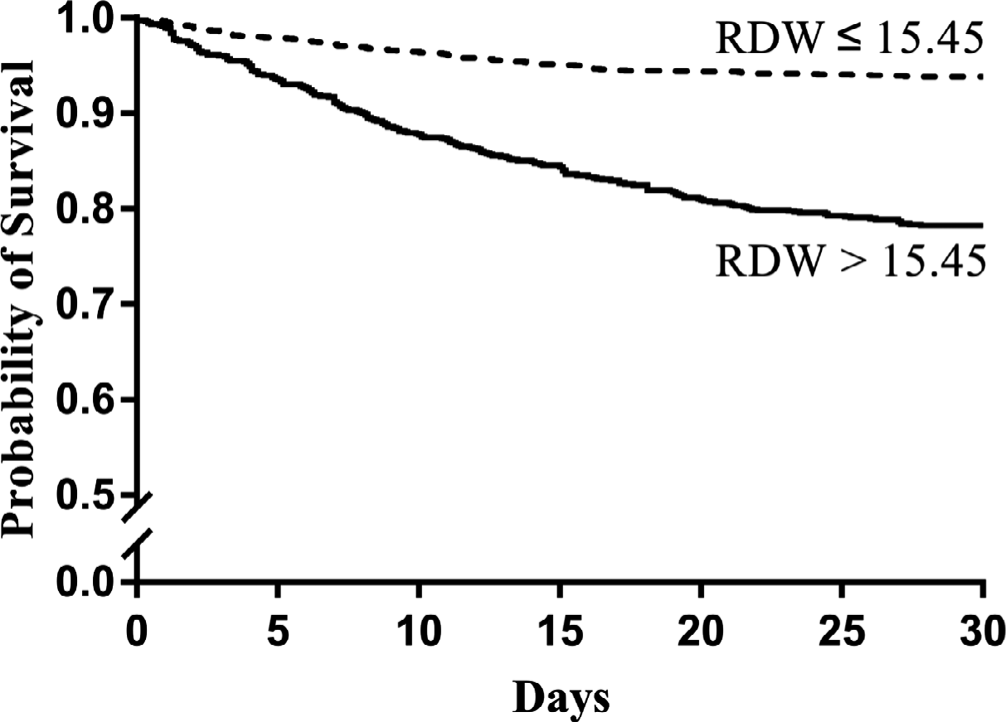
Kaplan–Meier survival for overall survival analysis of patients with high and low RDW.

The results of ROC curve analysis showed that the area under the curve of the 28‐day mortality of AUD patients predicted by RDW was 0.728 (95% CI: 0.700 to 0.755, *p* < 0.001; Fig. 1). The optimal cutoff value of RDW was 15.45%, with a sensitivity of 67.2% and a specificity of 67.3%. The baseline characteristics of patients in the low and high RDW groups are shown in Table 2. Length of stay in ICU, length of stay in hospital, in‐hospital mortality, and the 28‐day mortality in patients with high RDW values were significantly higher than those in patients with low RDW values (*p* < 0.001). Kaplan–Meier analysis showed that the 28‐day survival rate of AUD patients with high RDW values was significantly lower compared to those with low RDW values (*p* < 0.001; Fig. 2).

**Table 2:** Comparison of Clinical Characteristics of Patients With Different RDW Levels

	RDW ≤ 15.45 (*n* = 1,822)	RDW> 15.45 (*n* = 1,062)	*p* value
Age, year	51.3 (40.8 to 61.4)	54.3 (47.6 to 63)	<0.001
Gender, *n* (%)			<0.001
Female	387 (21.2%)	296 (27.9%)	
Male	1,435 (78.8)	766 (72.1%)	
Ethnicity, *n* (%)			0.043
White	1,286 (70.6%)	754 (71.9%)	
Black	124 (6.8%)	92 (8.7%)	
Others	412 (22.6%)	206 (19.4%)	
SBP, mm Hg	122.9 (111.2 to 135.6)	115.6 (105 to 129)	<0.001
Heart rate, beats/minute	88.3 (78.1 to 98.9)	89.9 (78.7 to 101.1)	0.090
Respiratory rate, beats/minute	18.5 (16.4 to 21.1)	18.5 (16.2 to 21.5)	0.954
Scoring systems			
SOFA	3.0 (1.0 to 5.0)	6.0 (4.0 to 9.0)	<0.001
SAPS II	27.0 (19.0 to 35.0)	36.0 (27.0 to 47.0)	<0.001
MELD	11.0 (7.0 to 20.0)	11.0 (8.0 to 21.0)	0.479
Comorbidities, *n* (%)			
Congestive heart failure	230 (12.6%)	168 (15.8%)	0.016
Hypertension	728 (40.0%)	405 (38.1%)	0.334
Liver disease	284 (15.6%)	686 (64.6%)	<0.001
Electrolyte disorders	570 (31.3%)	529 (49.8%)	<0.001
Coagulopathy	227 (12.5%)	399 (37.6%)	<0.001
Anemia	1,288 (70.7%)	1,008 (94.9%)	<0.001
Chronic pulmonary disease	270 (14.8%)	180 (16.9%)	0.128
Laboratory parameters			
WBC, 10^9^/l	11.9 (8.4 to 16.2)	10.6 (7.2 to 16.1)	<0.001
Hemoglobin, g/dl	11.6 (10.0 to 13.0)	9.2 (8.0 to 10.5)	<0.001
MCV, fl	93.0 (89.0 to 98.0)	95.0 (89.0 to 101.0)	<0.001
Platelet, 10^9^/l	181.0 (126.8 to 235.0)	105.0 (63.0 to 184.0)	<0.001
ALT, IU/l	104.0 (31.8 to 200.6)	44.0 (24.0 to 180.3)	<0.001
AST, IU/l	188.5 (45 to 331.3)	92.0 (48.0 to 331.3)	<0.001
Glucose, mg/dl	141.0 (116.0 to 181.0)	141.0 (114.0 to 184.0)	0.959
ICU LOS, day	2.6 (1.6 to 5.6)	3.1 (1.8 to 6.8)	<0.001
Hospital LOS, day	7.6 (4.0 to 13.7)	10.0 (5.1 to 18.7)	<0.001
In to hospital mortality, *n* (%)	103 (5.7%)	226 (21.3%)	<0.001
28 to day mortality, *n* (%)	113 (6.2%)	231 (21.8%)	<0.001

Continuous covariates are reported as median (interquartile range) and categorical variables as *n* (%).

ALT, alanine aminotransferase; AST, aspartate aminotransferase; ICU, intensive care unit; LOS, length of stay; MCV, mean corpuscular volume; MELD, Model for End to stage Liver Disease; RDW, red cell distribution width; SAPS II, Simplified Acute Physiology Score II; SBP, systolic blood pressure; SOFA, Sequential Organ Failure Assessment; WBC, white blood cell.

### RDW Is an Independent Risk Factor

The results of univariate and multivariate COX regression analyses are shown in Table 3. First, a univariate COX regression analysis was performed on potential risk factors, and then, statistically significant variables were included in the multivariate COX regression analysis (*p* < 0.05). Multivariate COX regression analysis showed that RDW (HR = 1.964, 95% CI: 1.429 to 2.698), ethnicity, SAPS II scores, liver disease, WBC, hemoglobin, and MCV were independent risk factors that affected the 28‐day mortality of AUD patients.

**Table 3:** Predictive Factors Affecting 28 to Day Mortality in Critically Ill Patients With AUD

	Univariate analysis		Multivariate analysis	
	HR (95% CI)	*p* value	HR (95% CI)	*p* value
RDW > 15.45%	3.804 (3.037 to 4.764)	<0.001	1.964 (1.429 to 2.698)	<0.001
Age	1.009 (1.006 to 1.012)	<0.001	1.005 (0.999 to 1.011)	0.087
Gender, male	0.909 (0.712 to 1.159)	0.442		
Ethnicity				
White	0.756 (0.594 to 0.962)	0.023	0.676 (0.518 to 0.881)	0.004
Black	0.472 (0.277 to 0.802)	0.006	0.382 (0.206 to 0.707)	0.002
Others	1 (Reference)			
SBP	0.972 (0.965 to 0.979)	<0.001	0.996 (0.988 to 1.004)	0.316
Heart rate	1.009 (1.003 to 1.016)	0.006	1.000 (0.993 to 1.007)	0.961
Respiratory rate	1.091 (1.066 to 1.115)	<0.001	1.027 (0.997 to 1.057)	0.074
SOFA	1.266 (1.239 to 1.294)	<0.001	1.025 (0.976 to 1.077)	0.326
SAPS II	1.071 (1.065 to 1.078)	<0.001	1.055 (1.042 to 1.068)	<0.001
MELD	0.994 (0.983 to 1.005)	0.274		
Congestive heart failure	1.353 (1.026 to 1.785)	0.032	1.059 (0.769 to 1.458)	0.727
Hypertension	0.934 (0.751 to 1.161)	0.537		
Liver disease	3.675 (2.951 to 4.577)	<0.001	1.569 (1.156 to 2.131)	0.004
Electrolyte disorders	2.454 (1.980 to 3.043)	<0.001	1.151 (0.892 to 1.483)	0.279
Coagulopathy	2.601 (2.097 to 3.226)	<0.001	0.994 (0.758 to 1.303)	0.964
Anemia	1.691 (1.240 to 2.307)	<0.001	0.856 (0.538 to 1.362)	0.512
Chronic pulmonary disease	1.164 (0.883 to 1.535)	0.281		
WBC	1.018 (1.014 to 1.022)	<0.001	1.008 (1.001 to 1.015)	0.029
Hemoglobin	0.887 (0.847 to 0.928)	<0.001	1.129 (1.048 to 1.215)	<0.001
MCV	1.068 (1.056 to 1.080)	<0.001	1.031 (1.018 to 1.044)	<0.001
Platelet	0.995 (0.994 to 0.997)	<0.001	1.000 (0.998 to 1.001)	0.874
ALT	1.000 (1.000 to 1.000)	0.966		
AST	1.000 (1.000 to 1.000)	0.019	1.000 (1.000 to 1.000)	0.896
Glucose	1.001 (0.000 to 1.002)	0.064		

AUD, alcohol use disorder; HR, hazard ratio; CI, confidence interval; RDW, red cell distribution width; SBP, systolic blood pressure; SOFA, Sequential Organ Failure Assessment; SAPS II, Simplified Acute Physiology Score II; MELD, Model for End to stage Liver Disease; WBC, white blood cell; MCV, mean corpuscular volume; ALT, alanine aminotransferase; AST, aspartate aminotransferase.

## Discussion

We found that the RDW of critically ill patients with AUD who died within 28 days of ICU admission was significantly higher than that recorded for survivors. A higher RDW (>15.45%) was associated with increased length of stay in ICU, length of stay in hospital, in‐hospital mortality, and 28‐day mortality. The COX regression analysis showed that an increase in RDW within 24 hours after ICU admission was an independent predictor of the 28‐day mortality in critically ill patients with AUD.

The mechanism of alcohol‐induced body damage is multifaceted, mainly involving alcohol metabolism, immune regulation, oxidative stress, and ion channel opening (Ceni et al., 2014; Fernandez‐Sola, 2020). Because of excessive systemic alcohol, AUD inpatients have a high risk and high severity of pneumonia, delayed wound healing, sepsis, and ARDS making these patients more likely to be admitted to the ICU (Joshi and Guidot, 2007; Moss et al., 2003; Silver et al., 2008). Therefore, it is important to explore the early risk factors of adverse events in AUD patients and implement timely intervention measures. However, reports analyzing prognostic risk factors for AUD patients are limited. Calvert and colleagues (2016) developed a clinical decision‐making system called AutoTriage based on measurement of 8 clinical variables that can be used to assess the risk of 12‐hour mortality in critically ill patients with AUD. The predictive ability of this system was found to be significantly better than the traditional MEWS, SAPS II, and SOFA scoring systems. Fuster and colleagues (2015) reported a cohort study of 909 AUD patients undergoing detoxification. At the median 3.8‐year follow‐up, the results showed that the presence of anemia was a predictor of midterm mortality in patients, while fibrinogen and ferritin levels were not related to midterm mortality.

An ideal prognostic marker would be easy to measure, fast, and cheap and can effectively predict mortality. RDW is a part of the complete blood cell count, reflecting the heterogeneity of the volume of red blood cells present. Because it can directly reflect the abnormalities in red blood cells, it is mainly used in the clinical diagnosis and monitoring of treatment for anemia. In 2007, Felker and colleagues (2007) initially reported that high RDW was highly predictive of poor prognosis in chronic heart failure. Since then, many studies have shown that high RDW is related to inflammatory response and oxidative stress, and can predict the severity and prognosis of various diseases, including cancer, cardiovascular disease, acute pancreatitis, and sepsis (Jo et al., 2013; Li et al., 2018; Parizadeh et al., 2019; Senol et al., 2013). Similarly, the current study showed that high RDW is a predictor of 28‐day mortality in critically ill patients with AUD. Erythrocytosis and megaloblastic anemia commonly occur with long‐term alcohol use, which can lead to increased RDW (Green and Dwyre, 2015).

Multiple mechanisms may play a role in the relationship between RDW and prognosis in critically ill patients with AUD. First, the alcohol metabolite, acetaldehyde, can increase the generation of free radicals, such as reactive oxygen and nitrogen, causing damage to cells or tissues (Wakabayashi, 2019; Yan and Zhao, 2020). Acetaldehyde affects the normal development of red blood cells, reduces their oxygen‐carrying capacity, and shortens the life of these cells (Waris et al., 2020). Second, the levels of tumor necrosis factor‐α, interleukin‐1β, and interleukin‐6 are increased in patients with AUD (Ansari et al., 2016). These inflammatory factors can affect hematopoietic function and hinder the maturation of red blood cells. Because immature red blood cells have larger volumes than mature ones, a high proportion of these immature cells can cause increased red blood cell heterogeneity and high RDW. Third, malnutrition, which is common in patients with AUD, can also lead to high RDW (Neuman et al., 2017; Salvagno et al., 2015).

A large sample size is one advantage of this study. However, the results should be interpreted carefully. First, this is a single‐center retrospective study based on a public database. Second, due to the different instruments and measurement techniques used to obtain RDW, the cutoff value of this study cannot be directly applied to other medical institutions. Third, this study only examined RDW within the first 24 hours and dynamic monitoring of changes in RDW may be a more meaningful parameter and this should be addressed in future studies.

In conclusion, to the best of our knowledge, this is the first study to report that high RDW is an independent predictor of short‐term mortality in critically ill patients with AUD. RDW is an easy‐to‐monitor, fast, and inexpensive indicator that may play a role in assessing potential risks and allows for adjusted treatment options in patients. Future, multicenter prospective research is needed to confirm the clinical value of RDW.

## Conflict of interest

The authors declare that there is no conflict of interests.

## Authors’ contributions

L.L. analyzed the data and wrote the article. L.P. designed the study protocol and drafted the manuscript. All authors approved for submission.
